# Early effect of bivalent human papillomavirus vaccination on cytology outcomes in cervical samples among young women in the Netherlands

**DOI:** 10.1002/cam4.5842

**Published:** 2023-03-25

**Authors:** Tessa M. Schurink‐van ’t Klooster, Albert G. Siebers, Joske Hoes, Folkert J. van Kemenade, Johannes Berkhof, Johannes A. Bogaards, Hester E. de Melker

**Affiliations:** ^1^ Department National Immunisation Programme, Centre for Infectious Disease Control National Institute for Public Health and the Environment Bilthoven The Netherlands; ^2^ PALGA Houten The Netherlands; ^3^ Department of Pathology Erasmus Medical Centre Rotterdam The Netherlands; ^4^ Department of Epidemiology and Data Science Amsterdam UMC, Vrije Universiteit Amsterdam Amsterdam The Netherlands

**Keywords:** bivalent HPV‐vaccine, cytological cervical abnormalities, high‐grade cervical lesions, high‐risk HPV, HSIL, human papillomavirus, low‐grade cervical lesions, LSIL

## Abstract

**Background:**

The first HPV‐vaccine eligible cohorts in the Netherlands will enter the cervical screening program in 2023. However, a substantial number of young women already have had a cervical sample taken before entry into the regular screening program. This study was initiated to explore early effects of HPV vaccination on detection of cytological abnormalities in cervical samples of women younger than the screening age.

**Methods:**

Results of cervical samples were obtained from the Dutch National Pathology Databank (PALGA) and were linked to the women's HPV vaccination status from the national vaccination registry (Praeventis) (*N* = 42,171). Occurrence of low‐grade and high‐grade squamous intraepithelial lesions or worse (LSIL and HSIL+) and high‐risk HPV positive tests (hrHPV) in the first cervical sample were compared between vaccinated and unvaccinated women by multivariable logistic regression analysis, corrected for age at cervical sampling and age of vaccination (12/13 years, ≥ = 14 years).

**Results:**

For fully vaccinated women (three‐ or two‐dose schedule), statistically significant reductions were seen for all outcomes compared to unvaccinated women (hrHPV: adjusted OR, 0.70, 95% CI, 0.63–0.79; LSIL: 0.70, 0.61–0.80; HSIL+: 0.39, 0.30–0.51).

**Conclusions:**

By linking nation‐wide registries on pathology and vaccination, we show significant beneficial early effects of HPV‐vaccination on LSIL, HSIL+, CIN3/AIS/carcinoma and hrHPV detection in young women upto 24 years of age who have a cervical sample taken before entry into the cervical cancer screening program.

## Graphical abstract

By linking nation‐wide registries on pathology and vaccination, marked reductions in low‐grade (LSIL) and high‐grade (HSIL) cytological cervical abnormalities were found in young women up to 24 years of age who were fully vaccinated against HPV and did have a cervical sample taken before entry into the cervical cancer screening program.

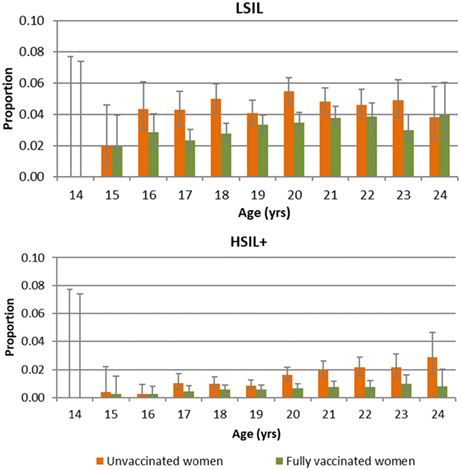

## 1 INTRODUCTION

Cervical cancer is the fourth most frequent cancer in women worldwide. The estimated number of new cases of cervical cancer in 2018 was 570,000. More than 311,000 women were estimated to die from cervical cancer.^1^ In the Netherlands, the incidence of cervical cancer has been increasing in recent years, reaching 9.9 per 100,000 women in 2019. Mortality from cervical cancer remains relatively stable with 2.5 deaths per 100,000 women in 2019.^2^

Cervical cancer is mostly caused by the sexually transmitted human papillomavirus (HPV).^1^ HPV infection is common in (young) sexually active women and men, that is, most persons will become infected at some point in their lives. Most HPV infections are cleared within 2 years, but when an infection with a high‐risk HPV (hrHPV) type persists, this can lead to the development of (precursor lesions of) cancer in the cervix or anogenital sites. Three vaccines are currently licensed in Europe for the prevention of HPV‐related diseases: a bivalent vaccine including the hrHPV types 16 and 18, a quadrivalent vaccine additionally including the low‐risk HPV types 6 and 11, predominantly associated with anogenital warts, and a nonavalent vaccine that covers 7 high‐risk types (HPV16/18/31/33/45/52/58), as well as the 2 low‐risk types HPV6 and HPV11.^3^

HPV vaccination for girls is performed within the context of the Dutch National Immunization Program (NIP). First, a catch‐up campaign for 13‐ to 16‐year‐old girls born between 1993 and 1996 was initiated in 2009. From 2010 onwards, girls were routinely offered HPV vaccination in the year they turned 13 (born in 1997 or later). Up to 2013, a three‐dose schedule of the bivalent HPV16/18 vaccine was used. In 2014, the schedule was changed to two doses of the bivalent vaccine.^4^ For girls born in 1993–1996, the vaccine coverage was 52%.^5^ For girls born from 1997 onwards, the vaccination coverage fluctuated between 46% and 63%.^6^

As a secondary prevention method, a nationally organized cervical cancer screening program was conducted in the Netherlands, for women between 30 and 60 years of age.^7^ The first birth cohorts who were eligible for HPV vaccination will enter the cervical cancer screening program in 2023. However, a substantial number of young women already have a cervical sample taken before entry into the regular screening program. Therefore, this study was initiated to explore the early effects of HPV vaccination on the detection of cytological abnormalities in cervical samples of women outside the screening program.

## 2 MATERIALS AND METHODS

### 2.1 Data sources and linkage

For this retrospective linkage study, we selected all Dutch women eligible for HPV vaccination (i.e., born from 1993 onwards) who have had a cervical sample taken between January 2009 and March 2018. Cytology and hrHPV test results (in case of co‐testing) were retrieved from the Dutch National Pathology Databank (PALGA).^8^ The HPV vaccination status of the women was retrieved from the national vaccination registry (Praeventis).

Data from both registrations were linked through pseudonymization by a trusted third party (ZorgTTP). Personal data was pseudonymized based on social security number (BSN) and surname (first eight characters), date of birth, and gender. About 94% of the cases matched one on one through this procedure and were included in the dataset. Cases with multiple matches were excluded from the dataset.

The procedure for linkage of the databases was assessed within the legal privacy framework and approved by both parties, that is, PALGA and Praeventis. Because this concerns retrospective research and therefore persons were not subjected to actions or rules of conduct, ethical approval was not necessary. There was an opt‐out procedure for both databases, which means that persons can exclude themselves from the database. Data were only used when the participant had not indicated that she objects against use of the data for scientific research, no additional written consent was needed for this purpose.

### 2.2 Cytological and high‐risk HPV testing

In the Netherlands, liquid‐based cytology (LBC) was used during the study period of interest. Cervical samples are coded according to the CISOE system, which can be easily translated into other coding systems. For this study, we translated CISOE to the Bethesda 2014 coding system with categories low‐grade squamous intraepithelial lesion (LSIL) and high‐grade squamous intraepithelial lesion or squamous cell carcinoma (HSIL+).^9^ The categories atypical squamous cells of undetermined significance (ASC‐US) and atypical squamous cells‐ cannot exclude HSIL (ASC‐H) were not taken into account in this study. Women with ASC‐US are not redirected to the gynecologist, because of the high chance of clearance. ASC‐H was not used in the Netherlands prior to 2017. Before that time, it would be signed out as HSIL or ASCUS.

For 20% of the women, co‐testing with cytology and hrHPV testing was performed. Information of the hrHPV genotypes was not recorded, so the result of the test was either hrHPV negative or hrHPV positive. Note that in the Netherlands, co‐testing is sometimes requested, but it is not recommended.

### 2.3 Inclusion and exclusion criteria

This study included all Dutch women eligible for HPV vaccination (i.e., born from 1993 onwards) who have had a cervical sample taken between January 2009 and March 2018. Women with missing data were not included in the analyses. In addition, some women were excluded from the analysis, that is, women from the youngest birth cohorts, that is, born in 2003 (*n* = 5) and 2004 (*n* = 7), and women above 24 years of age (*n* = 43), due to the very low numbers in these groups. Women who were vaccinated before the start of routine vaccination in 2009 (*n* = 350) and women who were vaccinated (*n* = 30) or eligible for vaccination after the first cervical sample had been taken (*n* = 4) were excluded as well.

### 2.4 Vaccination status

A woman was defined as fully vaccinated in case the woman received three doses of the HPV vaccine or two doses with a minimum of 5 months in between the doses when the first dose was administered before the 15th birthday. A woman was defined as partially vaccinated when she received one dose, two doses with less than 5 months in between, or two doses when started after the 15th birthday. If a woman received no HPV vaccine, then she was defined as unvaccinated.

### 2.5 Statistical analyses

Descriptive analyses, including chi‐square tests for differences according to vaccination status, were performed on the first cervical samples and the proportion of women with consecutive cervical samples taken within the study period.

Proportions of first cervical samples which were hrHPV positive, LSIL or HSIL+ were calculated by age for unvaccinated and fully vaccinated women, including 95% confidence intervals (95% CI) calculated with the Mid‐P method. The occurrence of LSIL, HSIL or worse (HSIL+) and hrHPV infection (in case of co‐testing) in the first cervical sample was compared between vaccinated and unvaccinated women by using multivariable logistic regression analysis, corrected for age at sampling (categorical), age group of vaccination (dichotomous: 12/13 years, catch‐up age) and birth cohort (categorical). In the analysis, vaccination status was stratified for fully vaccinated, partially vaccinated and unvaccinated women. In addition, analyses were stratified for age group of vaccination (12/13 years, catch‐up age, i.e., women in the regular campaign vs. women in the catch‐up campaign), because the effect of vaccination is expected to be highest in the regular campaign where almost all girls are vaccinated before sexual debut. In addition, women in the catch‐up campaign have a longer and identical length of follow‐up, while women in the regular campaign have a shorter and different time of follow‐up. Vaccine effectiveness (VE) was calculated by one‐adjusted OR for vaccinated versus unvaccinated women. For LSIL and HSIL+, this measure offers a good approximation of the relative reduction in risk of lesion occurrence, whereas for hrHPV, it offers a good approximation of the relative reduction in the hazard of repeated acquisitions.^10^, ^11^

In addition, the most severe diagnosis of all cervical samples from a women was analyzed. Both cytology and, if available, histology outcomes were taken into account to include all follow‐up outcomes. Histology outcomes were preferred over cytology, also in case of normal histology and abnormal cytology. Hereby, the occurrence of LSIL, HSIL, CIN1/CIN2/CIN‐NOS and CIN3/AIS/carcinoma was likewise compared between vaccinated and unvaccinated women by using multivariable logistic regression analysis, corrected for age at sampling (categorical), age of vaccination (12/13 years, catch‐up age) and birth cohort (categorical).

All statistical analyses were performed using SAS (V9.4, SAS Institute, USA).

## 3 RESULTS

### 3.1 Characteristics of cervical samples and study population

**FIGURE 1:**
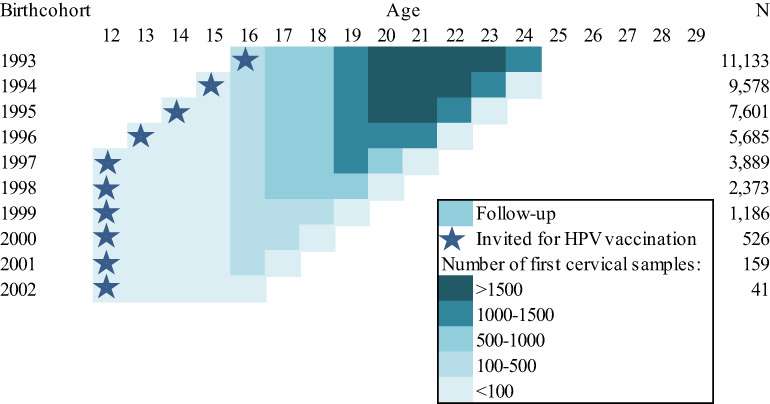
Follow‐up of the birth cohorts included in the final dataset and number of first cervical samples included by birth cohort (*N*_tot_ = 42,171).

The final dataset consisted of a total of 42,171 young women born between 1993 and 2002 (Figure 1). The follow‐up was the longest for birth cohorts 1993–1997, with follow‐up up to 10 years. For the youngest birth cohort, the follow‐up was only 3 years, that is, up to 16 years of age.

**FIGURE 2:**
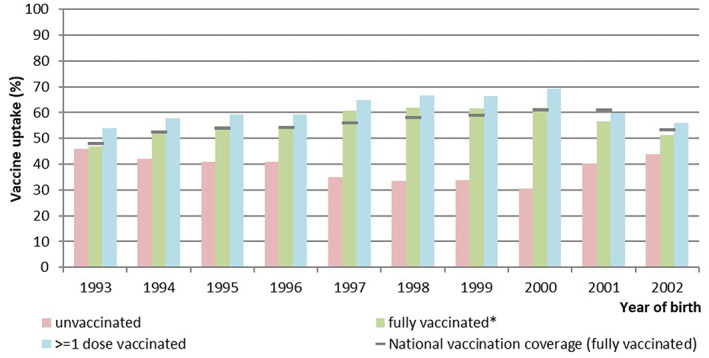
Vaccination uptake among the study population by birth cohort. *Three doses or two doses with a minimum of 5 months in between started before the 15th birthday.

The HPV vaccine uptake (fully vaccinated) among the study population ranged from 47% to 62% and is more or less comparable with the nationwide vaccination uptake among all women irrespective of the taking of cervical samples (Figure 2). The percentage of women in the population who had a cervical sample taken before age 25 was similar among fully vaccinated women (2.0; 95% CI, 2.0–2.1) and among unvaccinated women (2.0; 95% CI, 2.0–2.0).

More than 90% of the first cervical samples were taken because of (gynecological) complaints/symptoms (Table 1). The percentage of first cervical samples taken because of complaints/symptoms increased by age (82% for 14‐year‐olds to 99% for 24‐year‐olds). Overall, the reasons for taking the cervical sample were comparable for vaccinated and unvaccinated women. The percentage of first cervical samples with a co‐testing result also increased by age (14% for 14‐year‐olds to 38% for 24‐year‐olds). In unvaccinated women, co‐testing was slightly more common than in vaccinated women. Co‐testing was also more often performed when symptoms were reported (20.4% vs. 15.7%; *p* < 0.001).

**TABLE 1:** Reasons for the first cervical sample by vaccination status and type of tests.

	Unvaccinated		Fully vaccinated		Incompletely vaccinated	
	*n*	% (95% CI)	*n*	% (95% CI)	*n*	% (95% CI)
Cytology only	13,687	78.7 (78.1–79.3)	18,249	80.9 (80.4–81.4)	1823	81.6 (80.0–83.2)
Cytology and hrHPV test	3702	21.3 (20.7–21.9)	4300	19.1 (18.6–19.6)	410	18.4 (16.8–20.0)
**Reason cervical sample**						
Cytology only						
(gynecological) complaints/symptoms	12,416	90.7 (90.2–91.2)	16,627	91.1 (90.7–91.5)	1631	89.5 (88.0–90.8)
On own request	394	2.9 (2.6–3.2)	405	2.2 (2.0–2.4)	49	2.7 (2.0–3.5)
Indication/follow‐up research	114	0.8 (0.7–1.0)	142	0.8 (0.7–0.9)	17	0.9 (0.6–1.5)
Other	763	5.6 (5.2–6.0)	1075	5.9 (5.6–6.2)	126	6.9 (5.8–8.1)
Cytology and hrHPV test						
(gynecological) complaints/symptoms	3414	92.2 (91.3–93.1)	4040	94.0 (93.2–94.6)	383	93.4 (90.7–95.5)
On own request	108	2.9 (2.4–3.5)	76	1.8 (1.4–2.2)	8	2.0 (0.9–3.7)
Indication/follow‐up research	39	1.1 (0.8–1.4)	42	1.0 (0.7–1.3)	4	1.0 (0.3–2.3)
Other	141	3.8 (3.2–4.5)	142	3.3 (2.8–3.9)	15	3.7 (2.1–5.8)

The percentage of women who had more than one cervical sample taken within the study period was comparable for unvaccinated (29%, range 2–21 samples) and fully vaccinated (31%, range 2–18 samples) women (*p* = 0.156), although for incompletely vaccinated women the percentage of women with multiple cervical samples was slightly lower (26%, range 2–14 samples; *p* < 0.001).

### 3.2 Cytology results of the first cervical sample

**FIGURE 3:**
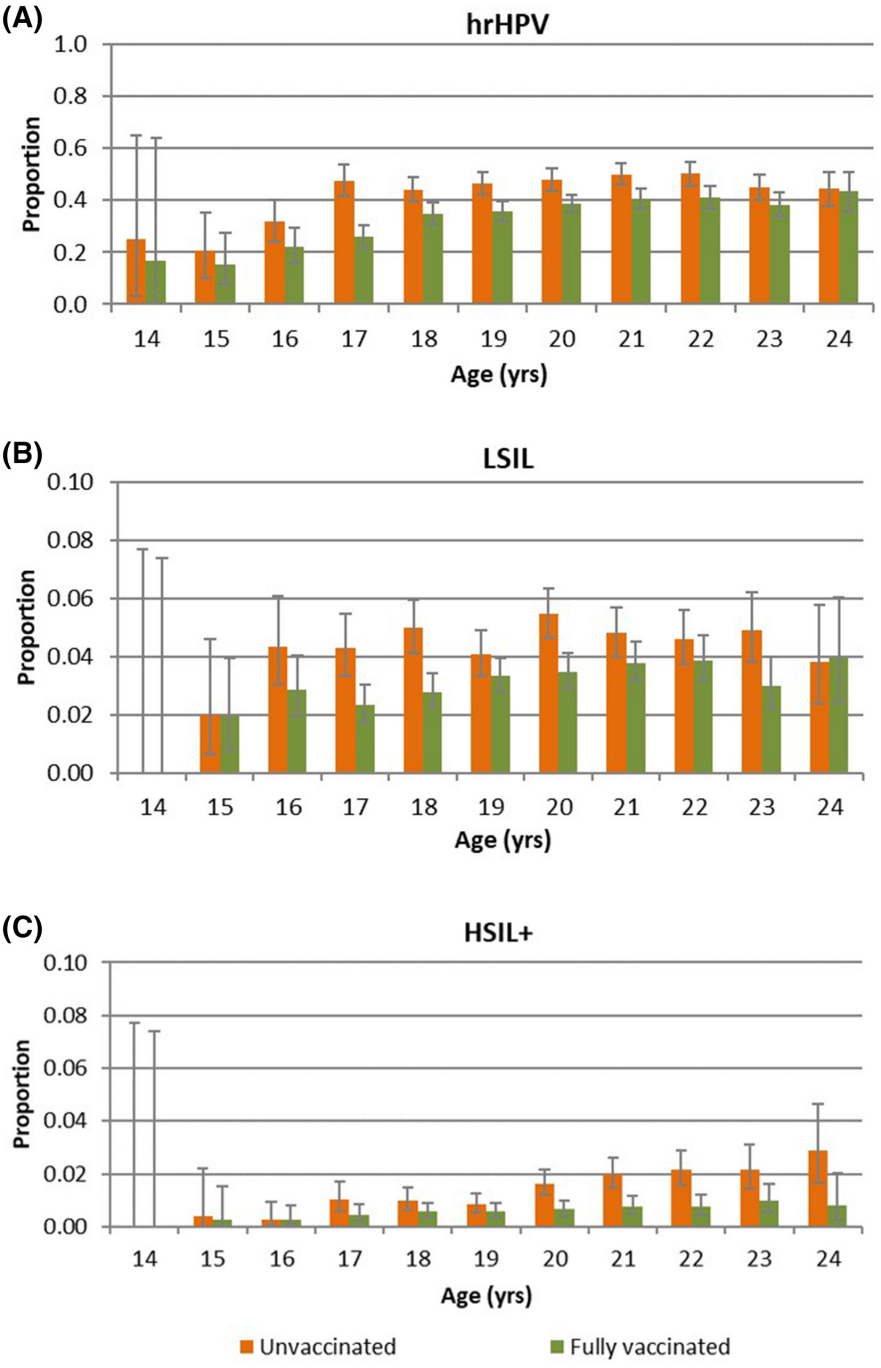
Proportion of first cervical samples positive for hrHPV (A), LSIL (B), or HSIL+ (C) by age for unvaccinated and fully vaccinated* women including 95% confidence interval (95% CI). *3 doses or 2 doses with a minimum of 5 months in between started before the 15th birthday.

The proportions LSIL and HSIL+ slightly increased with age (Figure 3B,C). Proportions of LSIL were higher in unvaccinated women than in fully vaccinated women from 16 upto 23 years of age. At 14, 15, and 24 years of age proportions were comparable. Proportions of HSIL were higher in unvaccinated women than in fully vaccinated women from 17 years of age.

Prevalence of abnormal cytology was lower in fully vaccinated women than in unvaccinated women (Table 2), that is, for LSIL OR, 0.70 (95% CI, 0.61–0.80; VE, 30.1% (20.1–38.7%)) and for HSIL+ OR 0.39 (95% CI, 0.30–0.51; VE, 60.8% (49.3–69.8%)). The association with HPV vaccination status was less strong among incompletely vaccinated women, that is, OR, 0.95 (95% CI, 0.76–1.20; VE, 4.6% (−20.0–24.1%)) for LSIL and OR 0.55 (95% CI, 0.34–0.88; VE, 45.5% (11.7–66.4%)) for HSIL+, although for HSIL+ not statistically significantly different from association with HPV vaccination status among fully vaccinated women (*p*‐value 0.18). In addition, no statistically significant differences were seen between ORs in women who were vaccinated at 12/13 years of age and women who were vaccinated at catch‐up age for both LSIL and HSIL+ (*p*‐values for fully vaccinated women: 0.19 for hrHPV, 0.47 for LSIL and 0.140 for HSIL+; *p*‐values for incompletely vaccinated women 0.69, 0.31, and 0.60, respectively).

**TABLE 2:** Positivity rates and logistic regression analyses for hrHPV, LSIL, and HSIL+ in first cervical samples.

	hrHPV		LSIL		HSIL+	
	Fully vaccinated (*N* = 4289)	Incompletely vaccinated (*N* = 409)	Fully vaccinated (*N* = 21,911^d^)	Incompletely vaccinated (*N* = 2180^d^)	Fully vaccinated (*N* = 21,911^d^)	Incompletely vaccinated (*N* = 2180^d^)
*N*	1562	160	712	94	140	19
Positivity rate, % (95% CI)^a^	36.4 (35.0–37.9)	39.1 (34.5–43.9)	3.2 (3.0–3.5)	4.3 (3.4–5.2)	0.6 (0.5–0.8)	0.9 (0.5–1.3)
Crude OR (95% CI)^a^	0.66 (0.61–0.73)	0.75 (0.60–0.92)	0.69 (0.62–0.76)	0.92 (0.74–1.15)	0.43 (0.35–0.53)	0.58 (0.37–0.93)
Adjusted^b^ OR (95% CI)^a^	0.70 (0.63–0.79)	0.78 (0.62–0.97)	0.70 (0.61–0.80)	0.95 (0.76–1.20)	0.39 (0.30–0.51)	0.55 (0.34–0.88)
Age group of vaccination: 12/13 years (*N* = 33,997)^c^, OR (95% CI)^a^	0.63 (0.56–0.72)	0.79 (0.56–1.11)	0.66 (0.57–0.77)	1.08 (0.76–1.53)	0.53 (0.39–0.74)	0.74 (0.30–1.83)
Age group of vaccination: catch‐up age (*N* = 8174)^c^, OR (95% CI)^a^	0.71 (0.63–0.80)	0.72 (0.55–0.94)	0.71 (0.62–0.81)	0.86 (0.65–1.13)	0.39 (0.30–0.51)	0.56 (0.32–0.96)

Abbreviations: CI, confidence interval; HSIL, high‐grade squamous intraepithelial lesion; hrHPV, high‐risk human papillomavirus; LSIL, low‐grade squamous intraepithelial lesion.

^a^ Reference = unvaccinated women positivity rate hrHPV 46.3% (44.7–47.9%; *n*/*N* = 1713/3698), LSIL 4.7% (4.3–5.0%; *n*/*N* = 788/16,928), HSIL+ 1.5% (1.3–1.7%; *n*/*N* = 251/16,928).

^b^ Corrected for age (categorical), age group of vaccination (12/13 years, > = 14 years) and birth cohort (categorical).

^c^ Corrected for age (categorical) and birth cohort (categorical).

^d^ Excluding samples which could not be properly assessed, that is, *n* = 638 fully vaccinated women and *n* = 53 incompletely vaccinated women.

### 3.3 hrHPV results of the first cervical sample

hrHPV positivity slightly increased with age, and was higher in unvaccinated women than in fully vaccinated women up to 23 years of age (Figure 3A). At 24 years of age, hrHPV positivity was comparable for unvaccinated and fully vaccinated women.

A statistically significantly different hrHPV positivity was seen in both fully vaccinated women (OR, 0.70 (0.63–0.79); VE, 30.1% (21.0–37.5%)) and incompletely vaccinated women (OR, 0.78 (0.62–0.97); VE, 21.9% (3.1–38.0%)) compared to unvaccinated women (Table 2). No statistically significant association with age group of vaccination (12/13 years or catch‐up age) was seen.

### 3.4 Most severe diagnosis of all cervical samples

For the most severe diagnosis of all cervical samples, statistically significant ORs were seen for LSIL and CIN3/AIS/carcinoma in fully vaccinated women, compared to unvaccinated women (Table 3). For HSIL and CIN1/CIN2/CIN‐NOS, ORs were not statistically significant reduced in fully vaccinated women. Among incompletely vaccinated women only a statistically significant reduction in most severe diagnosis was found for LSIL.

**TABLE 3:** Logistic regression analyses most severe diagnosis in all cervical samples.

	Fully vaccinated (*N* = 22,549)		Incompletely vaccinated (*N* = 2233)	
	*N*	aOR^b^ (95% CI)^a^	*N*	aOR^b^ (95% CI)^a^
LSIL	356	0.73 (0.61–0.89)	32	0.68 (0.47–0.99)
HSIL	34	0.60 (0.33–1.11)	9	1.82 (0.82–4.07)
CIN1/CIN2/CIN‐NOS	772	0.96 (0.80–1.16)	105	1.13 (0.80–1.61)
CIN3/AIS/carcinoma	52	0.28 (0.19–0.41)	14	0.60 (0.33–1.08)

Abbreviations: AIS, adenocarcinoma in situ; aOR, adjusted OR; CI, confidence interval; CIN, cervical intraepithelial neoplasia; HSIL, high‐grade squamous intraepithelial lesion; LSIL, low‐grade squamous intraepithelial lesion; NOS, not otherwise specified;

^a^ Reference = unvaccinated women (*N* = 17,389; N LSIL = 376; N HSIL = 35; N CIN1/CIN2/CIN‐NOS = 918; N CIN3/AIS/carcinoma = 184).

^b^ Corrected for age (categorical), age of vaccination (12/13 years, > = 14 years) and birth cohort (categorical).

## 4 DISCUSSION

With this nationwide retrospective linkage study we explored the early effects of HPV vaccination on cytological abnormalities in cervical samples of Dutch women up to 25 years of age. HPV vaccination coverage among these young women was comparable with the national vaccination coverage in these cohorts. By linking results of cervical testing and women's HPV vaccination status, we showed statistically significant reductions for hrHPV positivity, LSIL and HSIL+ in the first cervical samples of fully vaccinated women and for hrHPV positivity and HSIL+ in incompletely vaccinated women compared to unvaccinated women. Vaccinated women have had a cervical sample taken before the age of 25 just as often as unvaccinated women.

No statistically significant difference was seen between women vaccinated at 12/13 years of age and women vaccinated at catch‐up age, although ORs for LSIL and HSIL+ were slightly reduced for women vaccinated at 14 years or older than for women vaccinated at 12/13 years of age. It was expected that women vaccinated at younger age are better protected than women who were vaccinated at older ages. The observed difference might be caused by the shorter follow‐up period for women who were vaccinated at younger age (see Figure 1). However, compared to younger women we saw less differences in proportions of hrHPV and LSIL between 24‐year‐old unvaccinated women and fully vaccinated women. These women were vaccinated at 16 years of age, and some of them might have been exposed to hrHPV prior to vaccination. Possibly, a reduced effectiveness of vaccination due to prior exposure only becomes apparent from 16 years of age instead of > = 14 years, the stratification used in this analysis.

The VE found in this study for hrHPV can be compared with other Dutch studies in which the VE against HPV infections was estimated. A surveillance study in girls eligible for the catch‐up campaign (three vaccine doses) resulted in a VE against persistent hrHPV of 21.2% (95% CI, 2.5%–39.5%) up to 6 years following vaccination.^12^ In women visiting sexual health centers in the Netherlands, the pooled VE for type‐specific prevalent hrHPV infections was found to be 32.9% (95% CI, 20.2–43.7).^13^ Our study provides a comparable estimate against any‐type hrHPV infections (30.1%; 95% CI, 21.0–37.5%), though it is somewhat higher than the VE against combined hrHPV endpoints in the longitudinal surveillance study. However, in both surveillance studies highly sensitive tests were used to detect HPV whereas in our study only HPV‐tests validated for use in screening were used. This could explain the differences between the different studies. In addition, both surveillance studies found higher VE for vaccine types and cross‐protective HPV types. In our study, we were unable to distinguish between different HPV types.

The VE found in this study was more or less comparable with the international literature. In Denmark, in a birth cohort vaccinated with the quadrivalent HPV vaccine, the relative risk for HSIL was 0.6 (0.5–0.6) compared to an unvaccinated birth cohort.^14^ The Danish study found no effect of vaccination on ASC‐US or worse (RR 1.04; 0.96–1.12). The authors claimed that this difference might be due to the transition from conventional to LBC during the study period. In a nationwide Swedish cohort, a quadrivalent VE was found of 64% against histologically confirmed cervical intraepithelial neoplasia (CIN) Grade 2 or worse (CIN2+) for women vaccinated before 17 years of age.^15^ VE against CIN Grade 3 or worse (CIN3+) was found to be similar. A review by Arbyn et al. showed that in young women bivalent HPV vaccination reduced CIN2+ (RR 0.70; 95% CI, 0.58–0.85) and CIN3+ (RR 0.55; 95% CI, 0.43–0.71).^16^ A systematic review of the impact of the quadrivalent vaccine showed about 45% reduction for low‐grade cytological cervical abnormalities and about 85% reduction in high‐grade histologically proven cervical abnormalities.^17^ However, comparison with other countries is difficult because other countries have had a larger catch‐up vaccination campaign (including older ages), while regular screening starts at a younger age, that is, between 20 and 25 years of age.

The strengths of this study are the nationwide availability of the registry data and the individual linkage of cervical sample results and HPV vaccination status. Nevertheless, this study has also some limitations. The limitation of reliance on opportunistic screening results is that only a small proportion of the Dutch women have an opportunistic cervical sample taken (2%) of whom almost all had gynecological complaints/symptoms. Therefore, the women in this study are not representative for the general population of women in this age group. However, because this is the case for both vaccinated and unvaccinated women (see Table 1) we expect that the effect on the VE estimation is limited. In addition, the follow‐up of the women differs by birth cohort, depending on the age and year of vaccination, with a maximum of 10 years for those 24 years of age. Numbers of abnormalities, especially high‐grade lesions, are still very low in the women with shorter follow‐up (Figure 3). However, stratified analysis for women in the catch‐up campaign, that is, those with a longer and identical follow‐up time, showed comparable results as the overall results (Table 2). Moreover, information about dropout from the cohort due to emigration or death was lacking. This could have affected the results if cohort dropout was substantially different between vaccinated and unvaccinated women, which is not likely. Also, vaccinated and unvaccinated women might not be comparable in high‐risk behavior, which potentially could lead to bias. Unfortunately, we had no information on this matter. Finally, we were unable to distinguish between different HPV types and therefore unable to estimate the VE against HPV positivity rates/cytological abnormalities caused by vaccine types only. It is expected that the VE is higher for vaccine types than for hrHPV types.

The changes in the incidence of HPV types and precancerous cervical lesions due to the introduction of HPV vaccination will have consequences for the impact and efficiency of the cervical cancer screening program, starting at 30 years of age in the Netherlands. These are strongly determined by prevalence of pre‐cancerous lesions and the predictive value of a positive hrHPV test for underlying (malignant) lesions. As shown in this study, the vaccination has a higher effect on HSIL+ than on hrHPV‐positivity rates. This suggests that the predictive value of hrHPV positivity for underlying lesions will also decline in vaccinated women.

The vaccination uptake in the Netherlands fluctuated between 46% and 63%.^6^ This means that the target percentage of the WHO of 90% is not yet within reach.^18^ In 2022, vaccination of boys was added to the program in conjunction with a catch‐up campaign started for women and men up to and including 26 years of age. In addition, a renewed communication campaign started,^4^, ^19^ a decision tool is available, collaboration continued with the Dutch Cancer Society to draw attention to HPV vaccination, experience stories of people who have contracted HPV cancer are available online and there is more attention for reaching the group of people with a non‐Western migration background, who have a lower vaccination uptake.

In conclusion, by linking nation‐wide registries on pathology and vaccination, we were able to show significant beneficial early effects of HPV‐vaccination on hrHPV infection, LSIL, HSIL or worse and CIN3/AIS/carcinoma in young women up to 24 years of age who had a cervical sample taken before entry into the cervical cancer screening program.

## AUTHOR CONTRIBUTIONS

**Tessa Schurink‐van 't Klooster:** Formal analysis (lead); investigation (lead); methodology (equal); project administration (lead); visualization (lead); writing – original draft (lead). **Bert Siebers:** Conceptualization (equal); methodology (equal); resources (lead); writing – review and editing (equal). **Joske Hoes:** Writing – review and editing (equal). **Folkert J. van Kemenade:** Writing – review and editing (equal). **Johannes Berkhof:** Methodology (equal); supervision (equal); writing – review and editing (equal). **Johannes Bogaards:** Methodology (equal); writing – review and editing (equal). **H. E. de Melker:** Conceptualization (equal); methodology (equal); supervision (lead); writing – review and editing (equal).

## FUNDING INFORMATION

This research study was supported by the Ministry of Health, Welfare and Sport, and The Netherlands.

## CONFLICT OF INTEREST STATEMENT

The authors declare that they have no competing interests.

## SIGNIFICANT CONCLUSION(S) OR MESSAGE OF THE MANUSCRIPT

By linking nation‐wide registries on pathology and vaccination, marked reductions in low‐grade and high‐grade cytological cervical abnormalities were found in young women up to 24 years of age who were fully vaccinated against HPV and did have a cervical sample taken before entry into the cervical cancer screening program.

## Data Availability

Data sharing is not applicable to this article as no new data were created or analyzed in this study.
